# Assessment of Clinicians’ Satisfaction With Laboratories at a Public Sector Hospital in Karachi, Pakistan

**DOI:** 10.7759/cureus.5106

**Published:** 2019-07-09

**Authors:** Syed Arsalan Ahmed Naqvi, Aimel Hameed, Yusra Tanveer, Zahid Ali A Memon, Rida Masood

**Affiliations:** 1 Internal Medicine, Dow University of Health Sciences (DUHS), Karachi, PAK; 2 Surgery, Civil Hospital Karachi, Dow University of Health Sciences (DUHS), Karachi, PAK

**Keywords:** clinicians, laboratory, satisfaction, public sector, services

## Abstract

Objective

The goal of this study was to assess the level of satisfaction of clinicians regarding the provision of information, accessibility, and services by clinical laboratories at a public sector hospital in Karachi, Pakistan.

Methods

We conducted a cross-sectional survey of 151 participants from a public sector hospital in Karachi to assess their satisfaction regarding the associated laboratories. A five-point Likert scale questionnaire, consisting of 18 study items total, was used. Apart from the sociodemographics, the questionnaire was divided into three main sections: Services, Accessibility, and Provision of Information. The study lasted six months from October 2018 to March 2019.

Results

Most study participants were women. Less than one-third of the participants were consultants (21.9%). The overall satisfaction score was 62/90, indicating that the respondents were satisfied with most of the items on the scale. Factors such as notification about abnormal test results and courier services provided by the laboratories had the lowest satisfaction score.

Conclusion

Laboratory services are lacking in certain areas, specifically notifications and courier services, that need development and improvement, both of which can be achieved through seminars and clear communication between the laboratory staff and the associated clinicians.

## Introduction

Laboratories at hospitals are an essential component of healthcare services where clinical tests and investigations are used to assess patient health [1]. Laboratory reports contribute significantly to diagnostic decision making and patient management. Therefore, laboratory results must be accurate and reliable to ensure that treatment plans generate the best possible outcomes for patients [2].

Clinicians are considered the principal clients of laboratory services, and their level of satisfaction regarding the provided services is a prime quality measure in most quality assurance frameworks [3]. Evaluating client satisfaction with laboratory services is a major part of laboratory quality assurance programs and is a requirement for certification by the College of American Pathologists (CAP) and the Joint Commission on Accreditation for Healthcare Organizations [4].

Several previous surveys show that customer satisfaction with clinical laboratory services can be evaluated using a standardized survey tool. Some of the vital aspects covered in such surveys are the quality and legitimacy of test results, staff coordination, accessibility and responsiveness of laboratory management, test menu adequacy, lab courier services, and routine test turnaround time (TAT) [5].

The findings of the survey help assess physician satisfaction with the laboratory services provided and help identify the limitations of the laboratory. It serves as a guide for the laboratory to reflect on its lapses and limitations. The survey data should prompt the laboratory to constitute and implement measures through a proper action plan to ameliorate the quality of services and enhance its contribution to efficient and high-quality healthcare management. Therefore, this study assessed clinicians' satisfaction with the provision of information, accessibility, and services provided by clinical laboratories at a public sector hospital in Karachi, Pakistan.

## Materials and methods

This cross-sectional survey included 151 participants from a public sector hospital in Karachi. The study lasted six months from October 2018 to March 2019. We used a non-probability convenience sampling technique, and our sample size was 151 as calculated using OpenEpi software (Open Source Epidemiologic Statistics for Public Health) with a confidence interval of 95% with a 5% margin of error. This study included the practicing/on-duty general practitioners and clinicians from all departments in the hospital. Clinicians and general practitioners who were off-duty or who did not wish to participate in the study were not included. Intern or undergraduate medical students on training programs along with other healthcare providers (e.g., paramedical staff) were excluded from this study.

A self-administered systematically structured questionnaire, based on similar studies and surveys [2-3], was provided to the participants. The questionnaire consisted of a five-point Likert scale with 18 survey items in total (scoring: 1=very poor, 2=poor, 3=average, 4=good, 5=very good) and was composed of three major sections which included statements on services, accessibility, and information provided by the laboratories. All participants were informed of the contents of this study and provided informed written consent. The study was first piloted on 30 clinicians. The pilot sample was not included in the final analyses.

The responses recorded in the questionnaire were saved and coded. The data collected were entered in databases made through IBM SPSS Statistics for Windows, Version 24.0 (IBM Corp., Armonk, NY). Statistical analyses were obtained. Reliability analysis was done, and Cronbach’s alpha >0.7 was considered as reliable [6]. Means with standard deviations were measured for continuous variables while frequencies with percentages were computed for categorical variables. Mean scores were calculated for each item on the scale, and an overall score was computed to assess the relative satisfaction of the clinicians.

## Results

The study included 151 respondents, of which 55.6% (n=84) were women. Most respondents were residents (78.1%; n=118). Less than one-third of the respondents were consultants, including both consultant physicians (9.3%; n=14) and surgeons (12.6%; n=19). The mean duration of practicing in the hospital was 3.7 ± 3.7 years for clinicians. These sociodemographics are shown in Table 1.

**Table 1:** Sociodemographic variables

	Frequency (Percentage)
Males	67 (44.4%)
Females	84 (55.6%)
Consultant physicians	14 (9.3%)
Consultant surgeons	19 (12.6%)
Resident physicians	53 (35.1%)
Resident surgeons	65 (43%)
Mean duration of working in the hospital (years)	3.7 ± 3.7

The internal consistency statistics of the study item were reliable and done through Cronbach’s alpha (α=0.9).

The overall computed score for all the items in the study was 62/90 (69%), which showed relative satisfaction of the respondents over laboratories at the public sector hospital in Karachi. According to the study results in Table 2, the greatest satisfaction was observed in the laboratory reports being easy to read (3.80 ± 0.71) followed by the capacity of the laboratory to offer tests (3.66 ± 0.87) and the availability of the requested tests (3.65 ± 0.77). Other factors with higher satisfaction rates were the effectiveness of online results (3.63 ± 0.96), TAT of the test offered (3.63 ± 0.84) and accuracy of reference values (3.60 ± 0.74). A major dissatisfaction was the notification of the abnormal test results by the laboratory (2.91 ± 1) and courier services available from the laboratory (3.03 ± 1.27).

**Table 2:** Means and standard deviations of responses for study items

	Mean ± Standard deviation
Availability of the requested test	3.65 ± 0.77
Accuracy of the results reported	3.52 ± 0.73
Professionalism of the laboratory staff	3.49 ± 0.76
Technical services provided by the laboratory	3.50 ± 0.75
Complaint handling by the laboratory	3.35 ± 0.86
Capacity of the laboratory (maximum number of tests that can be offered by the lab at a single time)	3.66 ± 0.87
Courier services given by the laboratory	3.03 ± 1.27
Effectiveness of online results	3.63 ± 0.96
Turnaround time of the test offered	3.63 ± 0.84
Communication with the laboratory staff	3.36 ± 0.88
Conduct and cooperation of the laboratory staff	3.42 ± 0.84
Laboratory reports easy to report	3.80 ± 0.71
Availability of the pathologist/biochemist for queries	3.35 ± 0.84
Information on the availability of the tests at the laboratory	3.58 ± 0.78
Information on the proper sampling technique	3.32 ± 0.85
The accuracy of the reference values	3.60 ± 0.74
Request form clarity and extensiveness	3.38 ± 0.75
Notification about abnormal test results	2.91 ± 1
Total computed score	62.19/90
Score percentage	69.10%

The analysis also showed that male clinicians were relatively more satisfied than female clinicians (mean score, 3.52 for men, 3.41 for women). However, concerning position and designation at the hospital, the satisfaction over the laboratory was nearly the same as displayed in Table 3. Approximately 81% (n=123) of the respondents would recommend these laboratories, as shown in Table 4.

**Table 3:** Satisfaction scores with respect to gender and designation

	Mean ± standard deviation
Males	3.52 ± 0.51
Females	3.41 ± 0.48
Consultant physicians	3.41 ± 0.31
Consultant surgeons	3.47 ± 0.53
Resident physicians	3.43 ± 0.52
Resident surgeons	3.49 ± 0.50

**Table 4:** Responses on recommendation about the laboratory

Query	Response	Frequency (Percentage)
Would you recommend the laboratory?	Yes	123 (81.5%)
	No	28 (18.5%)

## Discussion

Clinical laboratory services are an everyday need for clinicians, and they should be reliable to avoid unnecessary challenges for the clinicians [1]. Several studies have assessed clinicians' level of satisfaction with laboratory services [7-10]. The objective of these studies was to analyze the strengths and limitations of laboratory services to take appropriate action to upgrade the quality of these services. To the best of our knowledge, no such research has yet been done in Karachi in a public sector hospital where the patient load is high, and subsequently, the frequency of laboratory tests ordered daily is also very high.

The overall satisfaction in our study was 69.1%, which was lower than the CAP Q-probes study in which the mean overall satisfaction score was 82% (4.1/5) [5]. Our satisfaction percentage was relatively lower than in the study conducted by Almatrafi et al. [7] in King Abdullah Medical City, Makkah, that was 73% (3.65/5). However, our findings were higher than those of the surveys conducted by Addis et al. [8] and Zaini et al. [9], in which the satisfaction was 51.5% and 53.3%, respectively. In this current study, approximately 81% of the clinicians would recommend the laboratories compared to the 96% to 100% recommendation level reported by the CAP Q-probes study [5].

In our study, the factor with the highest satisfaction rate was ease of readability of the laboratory reports (3.80). However, clinicians were least satisfied with the abnormal results notification (2.91) as compared to the study carried out by Almatrafi et al. [7], which had the highest satisfaction rate for critical results notification (3.8) while the least satisfying factors were specimen collection (3.39) and quality of test results (3.43). The low satisfaction over abnormal tests notification indicates that the pattern on which a report is filed is standardized, but there is a lack of effort in addressing the abnormal test results by laboratory staff. Hence, there is a need for more robust professionalism while addressing the abnormal test results.

The reliability and accuracy of the results reported are important factors for physicians [5]. This current study showed that respondents’ relative satisfaction with the accuracy of test results was similar to the findings reported by other studies [9,10]. Likewise, TAT is a key performance indicator for a laboratory [9]. The results of our study indicated a high level of satisfaction with the TAT. However, Jones et al. [5] reported that clinicians were least satisfied with the TAT (2.7). Similarly, the survey conducted at the Maternity and Children Hospital at Makkah showed that physicians were least satisfied with TAT [9].

Our study has a few limitations. Firstly, the sample size of our cross-sectional survey was small. Secondly, this study covered only a single public sector hospital and its associated laboratories in Karachi, so it does not represent the satisfaction state of the rest of the hospitals and their associated laboratories across the city. However, through this current study, we were able to assess the quality of laboratory services, accessibility, and provision of information by demonstrating the general satisfaction of clinicians. We identified that abnormal test result notification was the factor with the lowest satisfaction score among clinicians working in a public sector hospital in Karachi.

## Conclusions

The study showed that the clinicians working in a public sector hospital in Karachi showed relative satisfaction with the services, accessibility, and information provided by the associated laboratories. However, clinicians were least satisfied with the abnormal test result notifications and courier services provided by the laboratory. This could be addressed through dynamic communication between the laboratory staff and the concerned clinicians. There is a dire need for continuous development, considering the growing level of patient input at the hospital. Therefore, the results of this study should be disseminated through proper channels and forums. Furthermore, future studies should be conducted on a larger scale across Pakistan, given that it is a developing country. The Q-probes methodology should be used in both the public and private sector, so non-satisfactory elements in these service laboratories can be analyzed and improved.
